# Supplementary material for: WordCommentsAnalyzer: A windows software tool for qualitative research
Source: F1000Res. 2018 Sep 4;7:536. Originally published 2018 May 3. [Version 2] doi: 10.12688/f1000research.14819.2 (PMC6137411; doi:10.12688/f1000research.14819.2)
Supplement: Supplementary file 3 [file f1000research-7-17614-s0002.tgz › b2a79496-4ada-47c4-9682-a99ce867836d.zip!/Eight Iranian Health Journals/Archives of Rehabilitation_57.docx]

© Archives of Rehabilitation. This work is licensed under CC BY-NC 4.0 (https://creativecommons.org/licenses/by-nc/4.0/).

Volume & Issue: دوره 6 - شماره 3

Title: درد سینه عضلانی-اسکلتی در بیماران با ارزیابی قلبی طبیعی

http://rehabilitationj.uswr.ac.ir/article-1-75-fa.html

هدف: درد سینه عضلانی-اسکلتی یک تشخیص شایع بخشهای اورژانس و عمومی می‌باشد و واژه کوستوکندریت اغلب به جای آن و به عنوان مترادف استفاده می‌شود. تشخیص معمول آن توسط رد سایر علل و بیماریها است. ‌ارزیابی‌نقش‌دستگاه‌عضلانی‌- اسکلتی‌ در ‌بیماران ‌با ‌درد ‌قفسه‌ سینه‌ و ‌ارزیابی‌ نرمال قلبی هدف این مطالعه می‌باشد. روش بررسی: 971 بیمار که به علت آنژین صدری یا درد سینه ارجاع داده شده بودند، به طور آینده‌نگر بررسی شدند. ارزیابی‌های قلبی شامل: شرح حال، معاینه، ECG، Chest X Ray، اکوکاردیوگرافی، اسکن پرفیوژن و آنژیوگرافی در بیماران بر حسب مورد انجام شد. یکصد بیمار با علائم عضلانی-اسکلتی و ارزیابی‌های قلبی نرمال مورد بررسی قرار گرفتند. این گروه 100 نفری توسط متخصصین طب فیزیکی و توانبخشی مورد معاینه قرار گرفتند و شرح حال و معاینه و گرافی مهره‌های گردنی، نوار عصبی - عضلانی و آزمایشات لازم نیز برای آنها انجام شد. یافته‌ها: بر اساس تحقیق ما 3/10 درصد از بیماران با درد سینه دارای مشکلات عضلانی- اسکلتی بودند. از 100 بیمار با درد سینه عضلانی-اسکلتی و متوسط سن 2/43 ، 71 درصد آنها زن بودند . 38 درصد بیماران درد در ناحیه اسکاپولای چپ، و 60 درصد آنها نقاط حساس به درد، مخصوصاً در عضلات پشت گردن و اپی کندیل خارجی هومروس ‌داشتند‌ شیوع ‌EMG/NCS‌ پاتولوژیک 35 درصد‌ و‌ بیشتر ‌شامل ‌رادیکولوپاتی گردنی‌ بود. شیوع ‌پاتولوژیک‌ گرافی‌های ‌گردنی 26 درصد ‌و ‌بیشتر ‌شامل‌استئوآرتریت‌مهره‌های گردنی بود. نتیجه‌گیری: این مقاله علل درد سینه عضلانی-اسکلتی را مورد بررسی قرار می‌دهد و یک روش ارزیابی و پیگیری را معین می‌کند. نتایج، ارتباط غیرعلیتی را بین اختلال عملکرد سیستم عضلانی-اسکلتی و درد سینه آتیپیک نشان می‌دهند. یک معاینه دقیق شامل دامنه حرکتی ستون فقرات و لمس نقاط حساس باید انجام شود

Title: بررسی تأثیر برنامه روزانه مقیمان در مرکز اجتماع درمان‌مدار بر روابط اجتماعی آنان

http://rehabilitationj.uswr.ac.ir/article-1-76-fa.html

هدف: در این تحقیق، تاثیر برنامه روزانه مقیمان در مرکز اجتماع درمان مدار بر روابط اجتماعی آنان بررسی شد. از آنجائیکه انسان یک موجود اجتماعی است پس توانایی یادگیری هنجارها و ارزشهای فرهنگ خود را دارد، پس می‌آموزد که در تعامل با شبکه روابط اجتماعی اش چگونه برخورد نماید. روش بررسی: ترک اعتیاد به شیوه Therapeutic Community از جمله روشهای ترک اعتیاد می‌باشد که در آن به افراد مراجعه کننده به قصد ترک پایدار یا طولانی مدت مواد، آموزشهایی داده می‌شود. این آموزشها موجب تغییر گام به گام و موثر در افراد است که نگرش انان نسبت به امور مختلف از جمله مصرف مواد را تغییر می‌دهد. در این مطالعه، 30 نفر از افراد بعنوان نمونه مطالعه در معرض اجتماعی شدن مجدد (Resocialization) قرار گرفتند. یافته‌ها: در بررسی تاثیر برنامه روزانه مقمیان در مرکز اجتماع درمان مدار بر روابط اجتماعی آنان در خارج از مرکز نتایج به دست آمده نشان داد که آموزشهای ارائه شده در مرکز TC بر بهبود روابط اجتماعی افراد موثر واقع شده است، که میزان همبستگی 0/41 در سطح معنی داری 0/03 می باشد. یعنی کسانی که برای ترک پایدار مواد به مرکز مراجعه کرده اند روابط اجتماعی مناسبی را از خود نشان داده اند. این بهبود روابط زمانی بیشتر احساس می‌شود که فرد مشارکت بیشتری در برنامه های ارائه شده در مرکز داشته باشد. نتیجه‌گیری: این برنامه‌ها که به صور مختلف اعم از برنامه جلسات صبحگاهی (Morning Meeting)، کلاسهای روانشناسی، جلسات مشاوره فردی و گروهی و سایر برنامه‌های برگزار شده اجرا می‌گردد باعث می‌شود که فرد در فرایند اجتماعی شدن مجدد قرار گرفته و روابط اجتماعی اش بهبود یابد. بنابراین می توان چنین نتیجه گیری کرد که مرکز TC این توانایی را دارد که روابط اجتماعی افراد را بهبود بخشد ونحوه گذراندن یک زندگی اجتماعی توام با آرامش را به آنان بیاموزد. بنابر این بین میزان مشارکت افراد در برنامه های TC و بهبود روابط اجتماعی آنان در خارج از مرکز رابطه وجود دارد.

Title: مقایسه تأثیر هپارین وریدی با آسپرین خوراکی در 48 ساعت اول درمان در بیماران سکته ایسکمیک مغزی

http://rehabilitationj.uswr.ac.ir/article-1-77-fa.html

هدف: حوادث عروقی سیستم عصبی پس از سرطان و بیماریهای قلبی، سومین علت مرگ و میر در دنیا محسوب می‌گردد. یکی از راههای رایج درمان تجویز داروهای ترومبولیتیک است. اما این روش در حال حاضر در کشور ما قابل اجرا و در دسترس نیست. لذا تحقیقات بیشتر بر استفاده از داروهای ضد پلاکتی و ضد انعقادی معطوف شده است. بهمین منظور این مطالعه با هدف مقایسه اثرات آسپرین و هپارین در حوادث عروقی ایسکمیک ترومبوتیک مغزی در 48 ساعت اول وقوع استروک طراحی گردید. روش بررسی: در این مطالعه کارآزمایی بالینی بیمارانیکه در بخش اعصاب بیمارستان سینا همدان بستری بودند و شرایط ورود به مطالعه را داشتند به صورت تصادفی در یکی از دو گروه درمان با هپارین و درمان با آسپرین قرار گرفتند .در گروه هپارین 32 نفر و در گروه آسپرین 30 بیمار مورد بررسی قرار گرفتند. علائم بالینی بیماران در فرم مخصوص ثبت و درمان داروئی شروع شد. در گروه هپارین، ابتدا 100 واحد هپارین به ازاء هر کیلو گرم وزن بدن به صورت وریدی تزریق و سپس 1000 واحد در ساعت به صورت وریدی و به مدت 48 ساعت تجویز شد. در گروه آسپرین 325‌ میلی گرم آسپرین به صورت خوراکی یک بار در روز و به مدت دو روز تجویز شد. 48 ساعت پس از شروع درمان علائم بالینی بیماران مورد ارزیابی مجدد پزشک متخصص قرار گرفت و علائم در فرم مربوطه ثبت شد. اطلاعات جمع آوری شده با استفاده از نرم افزار spss و آزمون آماری مجذور کای آنالیز شد. یافته‌ها: در گروه هپارین علائم حسی %8/43 از بیماران بهبود نسبی داشته است و در گروه دریافت کننده آسپرین %3/33 افراد دارای بهبودی نسبی بوده اند (0/001=P). مقایسه میزان بهبودی ضعف عضلانی در دو گروه نشان می‌دهد که در گروه هپارین %3/56 از بیماران بهبود نسبی داشته اند ولیکن در گروه دریافت کننده آسپرین در %3/33 از بیماران علائم نشان‌دهنده بهبود نسبی بوده است (0/04=P). آزمون آماری تفاوت معنی داری را در بین دو گروه از نظر بهبود وضعیت تکلم نشان می‌دهد (0/01=P) اما وضعیت عملکرد اسفنگتر و وضعیت بینایی در دو گروه از نظر آماری با هم تفاوتی نداشته است. نتیجه‌گیری: نتایج پژوهش نشان می‌دهد که در هردو گروه علائم بالینی بیماران بهبود یافته است. اما درمقایسه دو گروه، در بیمارانیکه هپارین دریافت کرده‌اند میزان بهبود علائم بیشتر از گروه دریافت کننده آسپرین بوده است.

Title: بار خانوادگی و مشکلات مبتلایان به سندرم داون

http://rehabilitationj.uswr.ac.ir/article-1-78-fa.html

هدف: سندرم داون (تریزومی 21) شایعترین اختلال کروموزومی بوده و همراه با اختلالات مختلف جسمی، روانی، رفتاری، خانوادگی و اجتماعی است. پژوهش فوق با هدف بررسی مشکلات و بار خانوادگی سندرم داون به انجام رسیده است. روش بررسی: 61 خانواده بیماران ثبت شده در انجمن سندرم داون ایران در این بررسی شرکت نموده‌اند (31 خانواده صاحب پسر و 30 خانواده صاحب دختر). محدوده سنی بیماران 3 تا 17 سال بوده و از ابزار (پرسشنامه) دو مرحله ای که توسط گروهی متخصص شامل هفت محور (سه تا 6 گزینه‌ای) تهیه شده استفاده گردیده است. اطلاعات واصله در نرم افزار طراحی شده مخصوص جمع آوری و توسط spss دانشجویی مورد بررسی آماری قرار گرفت. یافته‌ها: بررسی فوق مشکلات عمده را در هفت گروه مشکلات جسمی، روانی و رفتاری، اجتماعی، آموزشی، نگهداری، حمایتی و ارتباطی می‌داند. متوسط نمره اولویت این مشکلات بسیار نزدیک به هم 19/72 (2/41=SD‌) از 20 بوده است. اکثر خانواده‌ها نیاز به آموزش را ضروری دانسته (%85) و تنها %10 آشنایی کافی با مراکز حمایتی و ارائه کننده خدمت دارند. %97 موارد از پذیرش خوبی در خانواده برخوردارند و در %96 موارد خانواده نگران فرزند بعد از مرگ خود می‌باشند. خانواده فرزند خود را علی‌رغم معلولیت مستعد دانسته و وی را در بهره‌گیری آموزشی توانا می‌داند. نتیجه‌گیری: بر اساس مطالعه ما کودکان مبتلا به سندرم داون در خانواده خود از پذیرش خوبی برخوردار بوده و سازگاری با اعضاء خانواده دارند. همچنین خانواده‌ها وی را سربار خانواده و یا مورد اختلاف در خانواده ندانسته و از داشتن وی سرافکنده نمی‌باشند. خانواده‌ها نیاز به اطلاع‌رسانی را ضروری دانسته، آموزش برای خود و فرزند خود را لازم و برنامه‌ریزی اجتماعی در جهت تأمین شغل و آینده فرزند را مهم می‌دانند.

Title: مقایسه نگرش به افراد معلول در خانواده‌های‌شان قبل و بعد از اجرای توان‌بخشی مبتنی‌بر جامعه در استان اردبیل

http://rehabilitationj.uswr.ac.ir/article-1-79-fa.html

هدف: پژوهش حاضر به منظور بررسی تاثیر پذیری اجرای طرح توانبخشی مبتنی بر جامعه به نحوه نگرش خانواده های دارای معلول روستایی استان اردبیل نسبت به معلولین و پدیده معلولیت اجراء شده است. برخی دیگر از پارامترهای دخیل در نحوه نگرش نسبت به معلولین از جمله سطوح تحصیلات، شهرستان محل اقامت، اجراء و یا عدم اجراء طرح توانبخشی مبتنی بر جامعه، شغل، سن، جنسیت و آشنایی با بهزیستی مورد لحاظ قرار گرفته است. روش بررسی: جامعه آماری پژوهش شامل کلیه خانوارهای روستایی استان اردبیل (اعم از روستاهایی که طرح مذکور در آنها اجراء نشده است و روستاهایی که طرح در آن اجراء شده است. در روستاهای شهرستانهای خلخال، نمین و نیر طرح اجراء شده است.) در سال 1383 می باشد که با استفاده از روش «نمونه برداری چند مرحله ای تصادفی» تعداد 173 خانوار و 346 نفر (به ازای هر خانوار دو نفر) در آن شرکت داده شدند. ابزار پژوهش، پرسشنامه محقق ساخته بود که علاوه بر بخش جمعیت شناختی، 31 سوال آن جهت سنجش نگرش خانواده ها بر اساس مقیاس لیکرت تنظیم شده بود. در پژوهش از روش «پیمایشی مقطعی» استفاده شده است. جهت تجزیه و تحلیل داده های استخراج شده، با استفاده از نرم افزار SPSS اقدام و در بخش توصیف اطلاعات از میانگین نمرات نگرش نسبت به معلولین، واریانس و انحراف استاندارد نمرات و در بخش تحلیل و استدلال از روش تحلیل واریانس یک طرفه یا یک راهه، آزمون پسین یا تعقبی شعفه و از آزمون t برای دو گروه مستقل استفاده شده است. یافته‌ها: نتایج پژوهش نشان داد در مناطقی که طرح توانبخشی مبتنی بر جامعه در آن اجراء شده است نگرش مطلوبتری در مقایسه با سایر مناطق نسبت به پدیده معلولیت وجود داشته است. همچنین برخی از متغیرها از جمله سطح تحصیلات، جنسیت و آشنایی با بهزیستی در نحوه نگرش نسبت به معلولیت تاثیرگذار بوده اند. سایر متغیرها در نحوه نگرش نسبت به معلولین تفاوت معنی داری را نشان نداده است. نتیجه‌گیری:‌ تأکید بر آموزش طرح توانبخشی، مبتنی بر جامعه و گسترش آموزش مهارتهای زندگی از مؤثرترین راههای افزایش کارایی و کیفیت زندگی معلولین می‌باشد که باید با جدیت دنبال شود.

Title: بررسی مقایسه‌ای شاخص‌های ثباتی دینامیک در افراد مبتلا به درد پاتلوفمورال

http://rehabilitationj.uswr.ac.ir/article-1-80-fa.html

هدف: کاهش حس عمقی درد و تغییر در الگوی فعالیت عضلانی در بیماران مبتلا به درد قدامی زانو دیده می‌شود. تعادل به اطلاعات سیستم های حس عمقی، بینایی، وستیبولار و حس پیکری وابستگی دارد . بنابراین این احتمال وجود دارد که بیماران مبتلا به درد قدامی زانو تعادل ضعیف تری داشته باشند. هدف از انجام این مطالعه بررسی تاثیر درد مفصل پاتلوفمورال برروی تعادل در افراد مبتلا به درد پاتلوفمورال ومقایسه وضعیت تعادل در افراد سالم و بیمار بود. شاخص های ثباتی اندازه گیری شده شامل قدامی- خلفی و طرفی بود. روش بررسی: مطالعه انجام شده غیرتجربی از نوع شاهدی ـ موردی در دو گروه 25 نفره افراد سالم و بیماران مبتلا به درد پاتلوفمورال بوده و هدف در این مرحله مقایسه متغیرهای مورد مطالعه در دو گروه افراد سالم و بیمار، همچنین یافتن مشکلات موجود در افراد بیمار در مقایسه با افراد سالم بود. روش نمونه‌گری غیراحتمالی از نوع نمونه‌گیری ساده، انتخاب شد. جهت ارزیابی تعادل از سیستم ثباتی بایودکس استفاده گردید و آزمون تعادلی ثبات دینامیک مورد استفاده قرار گرفت. ارزیابی از هر دو گروه سالم و بیمار صورت گرفت. یافته‌ها: مقادیر شاخص‌های تعادلی جهت حفظ تعادل بروی اندام مداخله نشان از وجود اختلاف معنی‌دار بین دو گروه بوده است. طوری که شاخص کلی تعادل، در حالت چشم باز و بسته، بین دو گروه دارای اختلاف معنی‌دار بوده است. اما شاخص های قدامی- خلفی و طرفی اختلافی را نشان ندادند. نتیجه‌گیری: از آنجا که در افراد مبتلا به درد پاتلوفمورال حس عمقی کاهش یافته و فعالیت عضلانی نیز تغییر کرده است این افراد از تعادل ضعیف تری برخوردارند وخصوصاً وقتی درک موقعیت فضایی توسط سیستم بینایی حذفشود تعادل آنها بیشتر مختل می‌شود.

Title: بررسی میزان استاندارد دیادوکوکاینتیک در بین دانش‌آموزان مقطع ابتدایی در سه منطقه 3، 8 و 19 شهر تهران

http://rehabilitationj.uswr.ac.ir/article-1-81-fa.html

هدف: جهت ارزیابی توانایی حرکت سریع و متغیر اندامهای گفتاری افراد از روش دیادکوکاینتیک استفاده می‌شود. دو روش برای بدست آوردن این معیار وجود دارد. در اولین روش فرد باید در زمان ثابت هجاهای مشخصی را سریع تولید نماید و در دومین روش فرد باید تعداد ثابت هجاهای مشخص را جهت تعیین زمان اجرای آن به صورت سریع تولید نماید. لذا این پژوهش با هدف بررسی میزان استاندارد دیادکوکاینتیک در بین دانش آموزان مقطع ابتدایی در سه منطقه 3، 8 و 19 شهر تهران انجام شده است. روش بررسی: جامعه مورد مطالعه در این تحقیق دانش آموزان مقطع ابتدایی سه منطقه آموزشی 3، 8 و 19 بودند که از میان آنها 1997 دانش آموز (980 نفر دختر و 1017 نفر پسر) به طور تصادفی انتخاب گردیدند. دانش آموزان باید در مدت 5 ثانیه هجاهای /پا/ ، /تا/ ، /کا/ و /پا تا کا/ را به صورت سریع تولید می‌نمودند و همچنین باید تعداد ثابت هجاهای مشخص را جهت تعیین زمان اجرای آن به صورت سریع تولید نمایدکه در این مطالعه دانش آموزان باید 15 دفعه هر یک از هجاهای فوق را تولید نمایند تا زمان آنها ثبت گردد. یافته‌ها: با بالارفتن پایه تحصیلی متوسط زمان تمام هجاها کاهش داشت و همچنین متوسط تعداد هجاها در زمان ثابت افزایش داشت. نتیجه‌گیری: نتایج حاصل از این تحقیق نشان می‌دهد که با افزایش سن توانایی میانگین تعداد تکرار هجاها افزایش می‌یابد و میانگین زمان تکرار هجاها کاهش می‌یابد. همچنین در این تحقیق ثابت گردید که بین میانگین تکرار هجاها در بین پسرها و دخترها تفاوت معنی داری وجود دارد.

Title: طبقه‌بندی بیماری‌های عصبی-عضلانی براساس معیارهای بالینی، تحلیل‌های ملکولی و ایمونوهیستوشیمی در بیماران ایرانی

http://rehabilitationj.uswr.ac.ir/article-1-82-fa.html

هدف: بیماریهای عصبی عضلانی یک گروه هتروژنوس از بیماریهای وراثتی است. بیش از 150 نوع از این گروه از بیماریها تاکنون شناسایی شده است. اگر چه اختلالات عضلانی کودکان یکی از علل اصلی ناتوانی می‌باشد، پیشرفتهای چشمگیری در زمینه علل ژنتیکی این بیماریها صورت گرفته است،که در زمینه پیشگیری و تشخیص آن بسیار مهم می‌باشد. معیارهایی که برای طبقه بندی این بیماریها استفاده می‌شود عبارتند از: سن بروز بیماری، میزان پیشرفت بیماری و نوع وراثت. بیماریهای عصبی عضلانی به چهار دسته طبقه بندی می‌شوند: میوپاتیها (بیماریهای دیستروفی عضلانی)؛ نوروپاتی‌ها (بیماری شارکوت ماری توث) بیماریهای محل اتصال عصب و عضله (سندرم میاستنی مادرزادی)، بیماریهای نورون حرکتی(آتروفی عضلانی نخاعی). هدف از این مطالعه طبقه بندی بیماریهای عصبی عضلانی بر اساس معیارهای بالینی و آنالیزهای مولکولی و ایمنو هیستو شیمی در بیماران ایرانی مراجعه کننده به مرکز تحقیقات ژنتیک است. از آنجایی که بیماریهای عصبی – عضلانی دومین معلولیت شایع می‌باشد لزوم بررسی بیشتر در مورد این گروه از بیماریها، در جمعیت ایران ضروری می‌باشد. بدین ترتیب این مطالعه برای اولین بار بر روی بیماران ایرانی صورت گرفت که پس از اخذ فرم رضایت نامه بررسی‌های ذکر شده انجام گردید. روش بررسی: در مجموع در این تحقیق 143 بیمار مشکوک به نقصهای عصبی عضلانی ارجاع شده به مرکز تحقیقات ژنتیک تحت معاینه بالینی و انجام آزمایشات آنزیم‌های عضلانی و الکترومیوگرافی قرار گرفتند و پس از آن بر حسب مورد آزمایشات ملکولی و ایمونوهیستوشیمی انجام گرفت. یافته‌ها: 82 بیمار با دیستروفی عضلانی میوتونیک، 19 بیمار با دیستروفی عضلانی دوشن و بکر، 6 بیمار دیستروفی میوتونیک مادرزادی (CMD)، سه بیمار FSHD، ده بیمار آتروفی عضلانی نخاعی (SMA)، دو بیمار دیستروفی میاستنی مادرزادی (CMS) و 21 بیمار مشکوک به دیستروفی عضلانی کمربند لگنی-شانه‌ای (LGMD) بودند. در مورد سایر موارد نیاز به بررسی مولکولی بیشتر و بررسی دقیق تر برحسب علایم بود که در این تحقیق جای نمی‌گرفت. نتیجه‌گیری: در مواردی که دیستروفی میوتونیک نوع یک علیرغم ظن بالینی تایید نگردید، بررسی نوع دو دیستروفی میوتونیک ضروری می‌باشد. در بیماران مبتلا به دیستروفی عضلانی کمربند لگنی- شانه ای که 5 آنتی بادی مورد بررسی طبیعی بودند. بررسی آنالیز Multiplex western-blot توصیه می گردد. همچنین برای موارد دیستروفی عضلانی مادرزادی غیر از بررسی مروزین،‌ بررسی سایر پروتئین های درگیر و نیز انجام آزمایشات ملکولی جهت تعیین موتاسیون لازم می‌باشد.

Title: بررسی تأثیر زمان جراحی و ارجاع به توان‌بخشی بر نتایج ترمیم تاندون‌های فلکسوری زون 2

http://rehabilitationj.uswr.ac.ir/article-1-83-fa.html

هدف: با توجه به اینکه درمان توانبخشی با حرکت اکتیو زودهنگام پس از ترمیم تاندونهای فلکسوری زون 2 به عنوان یک روش مناسب جهت ترمیم‌های این ناحیه شناخته شده است بررسی سایر عوامل مثل زمان به کارگیری استرس، نحوه استرس و زمان ارجاع بیمار پس از جراحی و اثر زمان جراحی پس از آسیب، بر نتایج لازم است. روش بررسی: بررسی حاضر روی 57 بیمار که آسیب تاندونهای فلکسوری زون 2 داشتند و همگی به روش 4-Strand ترمیم شده و به مرکز توانبخشی دست مراجعه کرده بودند انجام شد. همه بیماران به روش حرکت اکتیو زودهنگام درمان شدند. در انتهای هفته هشتم همه بیماران از نظر دامنه حرکت، حرکت اکتیو کلی انگشتان، فلکشن کانترکچر مفصل PIP و فلکشن گپ تحت ارزیابی قرار گرفتند مشخص شد. یافته‌ها: با ارزیابی آنالیز واریانس یک طرفه نوع انگشت آسیب دیده، جنس، دست آسیب دیده اثری روی نتایج نداشت. زمان ارجاع با  0/07=P رابطه معناداری با حرکت اکتیو کلی انگشتان (T.A.M) داشت که بهترین زمان ارجاع کمتر از یک هفته است. فاصله بین آسیب تا جراحی با 0/06=P رابطه مستقیم با حرکت اکتیو کلی انگشتان داشتند. بیشترین T.A.M در ترمیم‌های اولیه (10 روز اول پس از آسیب) دیده شد. هرچه جراحی با تأخیر بیشتر انجام شده بود. فلکشن کانترکچر مفصل PIP بیشتر دیده شد. نتیجه‌گیری: با ترمیم اولیه (10 روز اول پس از آسیب) و ارجاع به موقع و زود به توانبخشی (کمتر از یک هفته) نتایج ترمیم‌های زون 2 بهتر خواهد شد.
